# Drosophila as a Model to Understand Second Heart Field Development

**DOI:** 10.3390/jcdd10120494

**Published:** 2023-12-12

**Authors:** Cayleen Bileckyj, Brenna Blotz, Richard M. Cripps

**Affiliations:** Department of Biology, San Diego State University, San Diego, CA 92182, USA

**Keywords:** Drosophila, heart, second heart field, DiGeorge syndrome, development

## Abstract

The genetic model system Drosophila has contributed fundamentally to our understanding of mammalian heart specification, development, and congenital heart disease. The relatively simple Drosophila heart is a linear muscular tube that is specified and develops in the embryo and persists throughout the life of the animal. It functions at all stages to circulate hemolymph within the open circulatory system of the body. During Drosophila metamorphosis, the cardiac tube is remodeled, and a new layer of muscle fibers spreads over the ventral surface of the heart to form the ventral longitudinal muscles. The formation of these fibers depends critically upon genes known to be necessary for mammalian second heart field (SHF) formation. Here, we review the prior contributions of the Drosophila system to the understanding of heart development and disease, discuss the importance of the SHF to mammalian heart development and disease, and then discuss how the ventral longitudinal adult cardiac muscles can serve as a novel model for understanding SHF development and disease.

## 1. Importance of Understanding Heart Development

Due to the pervasive nature of heart disease in the population, the ability to identify, understand, and treat cardiac-related health problems is of extreme importance. Congenital heart defects (CHDs) are one of the most common defects that occur during fetal development, with an approximate birth prevalence rate of 17.9 per 1000 births globally in 2017 [1]. Additionally, heart disease is the leading cause of mortality in the United States, where cardiovascular disease (CVD) was the cause of 928,741 deaths in 2020 (AHA Heart Disease and Stroke Statistics—2023 Update). Given that congenital conditions arise through departures from normal development, pediatric cardiologists are also cardiac developmental biologists who are interested in the formative processes contributing to the developing heart.

## 2. Development of the Embryonic Drosophila Heart

Model organisms, such as fruit flies, zebrafish, chicks, frogs and mice, have played instrumental roles in unraveling the mechanisms of heart development and disease in humans due to the discovery that conserved transcriptional and signaling pathways act to promote heart development in these highly different organisms [2,3,4,5,6,7,8,9].

*Drosophila melanogaster* (fruit flies) is a model organism that has been vital in defining the intricate genetic mechanisms governing human heart development. Their primitive heart, referred to as the dorsal vessel, consists of a linear cardiac tube spanning most of the length of the body. The posterior portion is referred to as the heart, and the anterior portion is termed the aorta [10]. The formation of this rudimentary heart begins early on in Drosophila development (reviewed in [11]). After gastrulation, the mesoderm in each body segment is divided into four distinct quadrants by expression in the overlying ectoderm of the signaling molecules Dpp (orthologous to BMP) and Wingless (orthologous to mammalian WNTs). These quadrants give rise to visceral muscle, heart, fat body, or skeletal muscle depending on whether they receive either, or both, or none, of these signals [12,13,14,15,16,17].

The bilateral dorsal mesodermal cells that come into contact with both ectodermal BMP and WNT signals acquire cardiac fate [12,17]. These pathways sustain the expression of the cardiogenic gene *tinman* (*tin*), which encodes a transcription factor necessary for heart and visceral muscle specification [12,18,19,20]. The GATA factor gene *pannier* (*pnr*), along with *tin*, the *Dorsocross1-3* T-box genes (*Doc1-3*) and the *H15/midline* T-box genes, are required for the differentiation of cardial cells [21,22,23,24]. In addition, expression of the MADS domain transcription factor Myocyte enhancer factor-2 (MEF2) is activated by, and then works in partnership with, Tin and Pnr [21,25,26] to promote cardiac differentiation [27].

**Figure 1:**
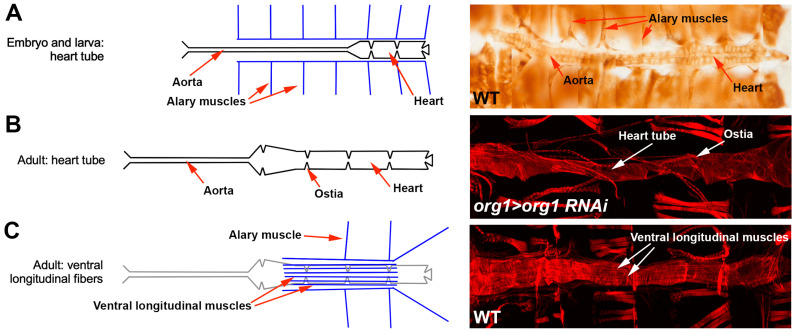
Organization of the Drosophila heart during development. Left: Diagrams of heart structure; right: images of the heart from different stages of development. Anterior is to the left. (**A**): The wild-type (WT) embryonic and larval structure comprises a posterior heart and an anterior aorta supported by alary muscles. (**B**): At the adult stage, the larval aorta has been remodeled into a robust heart tube perforated by ostia. Heart of a knockdown lacking ventral longitudinal muscles is shown to visualize the heart tube. (**C**): Wild-type adult heart showing ventral longitudinal muscles. Adult photomicrographs show the heart only and not the aorta.

By the end of embryogenesis, bilateral cardiac precursor cells migrate to the dorsal midline to form a cardiac tube (Figure 1A). The muscular portion of the tube comprises *tin*-expressing cardiomyocytes plus smaller cells that express the orphan nuclear receptor gene *seven-up* (*svp*), which represses the expression of *tin* [28,29]. Additionally, pericardial cells contribute nephrocyte-like functions [30].

The *svp*-expressing cells in the posterior heart form inflow tracts called ostia, while those in the aorta form part of the cardiac wall. During pupal metamorphosis, most of the posterior region of the cardiac tube, corresponding to the heart, is histolyzed. Aorta cells differentiate into the adult heart, and the *svp*-expressing cells in the aorta are modified into ostia [28].

The dorsal vessel is supported by seven pairs of alary muscles, which are located along the dorsal vessel and attaching near the *svp*-expressing pericardial cells [31,32]. These skeletal muscles develop under the control of the mammalian *Tbx1* ortholog *org-1*, and the Hox genes *Ubx* and *abd-A* directly control normal alary muscle patterning [32,33]. While at least three pairs of alary muscles are present in adult Drosophila as well, the anterior alary muscles dedifferentiate into alary muscle-derived cells (AMDCs) that give rise to the ventral longitudinal muscles (VLMs) of the adult heart [34,35] (Figure 1B,C). Alary muscles provide support to the dorsal vessel and aid in the circulation of hemolymph [10,31]. The absence of alary muscles in pupae causes an inability of the heart lumen to open, thus decreasing heart systolic and diastolic phases but leaving heart rhythm unaffected; this indicates alary muscles are not required for the heart to beat, but they are likely necessary for the heart to function correctly [36].

## 3. Parallels with Vertebrate Heart Development and Specification

Although the anatomical structure of the Drosophila heart differs from that of mammals, it is now broadly appreciated that the genes functioning to fashion the Drosophila heart represent a core of the transcription factors and signaling pathways that are necessary for heart formation across the animal kingdom. It is important to note that in general, the Drosophila genome does not have the genetic redundancy characteristic of vertebrate genomes, where single Drosophila genes (such as *Mef2*) have four close vertebrate orthologs (*mef2a-d*). This has enabled the rapid elucidation of gene function in cardiogenesis using Drosophila. In vertebrates, the presence of multiple paralogs for any cardiac regulatory factor can allow for redundancy in gene function and also enable subfunctionalization.

Some of the relevant conserved cardiogenic transcription factors include the following: *Nkx2.5* (orthologous to Drosophila Tin [37]), GATA factors including GATA4 (orthologous to Pnr [38,39]), Tbx20 (orthologous to H15/mid [40,41,42,43]), Tbx5 (orthologous to Doc1-3 [44]); MEF2A-D (orthologous to MEF2 [45,46]), and Tbx1 (orthologous to Org-1 [47]). Mammalian Hand1/2 are also necessary for heart development [48,49], and while it is unclear if a Drosophila ortholog contributes significantly to embryonic heart development, Drosophila Hand appears to be essential for normal adult heart patterning [50,51].

Conserved signaling pathways for cardiogenesis include BMP signaling (orthologous to Dpp [52]) and Wnt signaling (orthologous to Wg [53,54]). The contributions of many of these factors to cardiogenesis were first characterized in the fly system and subsequently shown to function during vertebrate heart development.

Since 75% of human disease genes have fly orthologs [55], this conservation of genes allows Drosophila to not only serve as a model to elucidate the mechanisms of human development but also act as a model for human disease [56]. The short life cycle of flies, and the ready ability to manipulate gene expression in this system using RNAi [57], has made Drosophila an ideal organism to screen for candidate disease genes [58,59]. For example, Ekure et al. performed the exome sequencing of 98 Nigerian children suffering from CHD and their unaffected parents. Several de novo mutations were identified as candidate contributors to CHD. To rapidly determine which mutations were most likely to be causative, the expression of genes of high interest were knocked down in the Drosophila heart tube by crossing a *Hand–Gal4* line to *UAS–RNAi* lines for each gene under investigation. A *Hand–GFP* marker was used to visualize if any cardiac defects occurred. Their data identified nine genes, four of which were not previously associated with CHD, that caused significant mortality in Drosophila, thereby identifying new putative human CHD genes [60].

Drosophila can also be used to quickly evaluate the pathogenicity of variants of unknown significance. Lovato et al. [61] modeled a human *NKX2-5* variant of unknown significance, K158N, in Drosophila *tin* (Tin^R321N^). They demonstrated that the mutant protein was deficient in vitro in gene activation and in physical interaction with the Tbx5 ortholog Dorsocross 1. Moreover, an in vivo CRISPR-induced mutation in *tin* mimicking the human variant resulted in minor but significant defects in the adult heart structure.

Clearly, Drosophila can be used as an efficient system to identify genes and mechanisms of human cardiac disease. Nevertheless, the mammalian heart is a highly complex organ that undergoes significant remodeling during development, including cardiac morphogenesis and the addition of a second group of cardial cells termed the second heart field (SHF), as discussed below. Can Drosophila also contribute to our knowledge of these more complex processes?

## 4. Discovery and Importance of the Second Heart Field

Research into early heart development and differentiation started many decades ago, often taking advantage of the highly conserved chick model. In the 1940s, histological analysis of chick blastoderm-derived grafts revealed two areas with heart-forming potency, which was the first experiment to confirm the existence of bilateral heart precursors [62].

After the fusion of the bilateral plates, a linear heart tube with an atrial and venous pole goes through a process known as looping morphogenesis, which is also known as cardiac dextral looping. This is a process of rightward looping [63] that forms the helical heart musculature required for vertebrate double circulation. It was subsequently found that precardiac mesodermal cells contribute to the growing heart tube during this looping period [64]. The outflow tract of the heart, the conduit that passes blood connecting the embryonic ventricles of the heart to the aortic sac, forms following the initiation of looping [65]. In the early 2000s, fate mapping and tissue ablation studies revealed a new population of cells in addition to the bilateral heart field progenitors that contribute to the outflow tract and right ventricle. This multipotent population of cells was termed the anterior (or secondary) heart field (SHF; [66]). In the same year, a mouse line with a *lacZ* transgene integrated upstream of the Fibroblast Growth Factor 10 (*Fgf10*) locus was created, and it was observed that *Fgf10* expression marked the splanchnic mesodermal precursors that make up the so-called second heart field (SHF) [67].

LIM domain homeobox gene *Islet-1* (*Isl1*) is a well-known neuronal specification marker that is expressed in early cardiac progenitor cells and the underlying endoderm in the chick embryo. The first heart field (FHF) cells, the first mesodermal cells that differentiate into the heart tube, down-regulate the expression of *Isl1*, whose expression persists in the SHF [68]. In *Isl1* homozygous null mice, embryonic hearts were severely underdeveloped and failed to undergo looping. A combination of Cre-recombinase lineage tracing and BrdU labeling revealed that cells expressing *Isl1* make up the majority of cells in the outflow tract, right ventricle, and atria as well as parts of the left ventricle. Additionally, *Isl1* is necessary for the proliferation and survival of SHF cells of the pharyngeal foregut endoderm and splanchnic mesoderm [69]. This study further bolstered the idea that second heart field cells are the cardiac progenitors that lead to the formation of the outflow tract and right ventricle, and it provided an explanation for the cardiac defects observed in *Isl1−/−* embryos. Cell fate mapping studies have enabled us to understand that second heart field cells contribute to the poles of the elongating heart tube during looping morphogenesis to form the myocardium, smooth muscle, and endothelial cells that ultimately contribute to the outflow tract, right ventricle, and part of the atria [70,71], as reviewed in Kelly [72].

Also important in the contribution of the SHF to the outflow tracts are cardiac neural crest cells (CNCCs), which are a temporary cell population that functions in early development for the formation of arteries as well as cardiac septa and valves [73]. The abnormal development of CNCCs is associated with several congenital heart defects [74].

Overall, the formation of the heart is a complex process that integrates contributions of cells from multiple different lineages to generate the functional organ.

## 5. Genetic Programs for Development and Signaling between the Heart Fields

Since the discovery of the SHF, significant effort has been expended to understand the specification of this structure and how it contributes to the formation of the mature heart in collaboration with the FHF.

In addition to Isl1, several other transcription factors are now known to function specifically within the SHF to promote its development, not the least of which is cardiac homeobox factor Nkx2.5, which is detected in both the primary and secondary heart fields [75]. A number of roles for *Nkx2.5* in early cardiac specification have been proposed, including the activation of a Bmp2/Smad negative feedback loop resulting in controlled cardiomyocyte proliferation and the contribution of SHF cells to the developing heart tube [76]. *Nkx2.5* is needed cell autonomously in the mesoderm for SHF formation, since *Nkx2.5* null mouse embryos, as well as mesodermal deletion mutants exhibit phenotypes of disturbed SHF development, while murine *Nkx2.5* endodermal deletion mutants develop normally [77].

A major discovery in SHF development occurred in 2002 when loss of function experiments revealed that disease-associated T-box transcription factor Tbx1 plays a role in the formation of the SHF. Tbx1 is necessary for cell contribution to the outflow tract, and it is required for the recruitment of SHF cells necessary for proper OFT alignment and truncal valve septation. A luciferase reporter assay in the same paper revealed that SHF-associated gene *FGF10* is a direct target of Tbx1, and a deletional mutant study revealed a genetic interaction between *Tbx1* and *FGF8* during the development of the aortic arch [78]. These and other experiments suggest that Tbx1 also plays a non-autonomous role in SHF cell proliferation [79]. Reinforcing this idea, Forkhead (Fox) proteins are involved in *Tbx1* transcriptional regulation through enhancer regions containing a binding site for Fox transcription factors [80]. The molecular function of *Tbx1* in OFT development is still an emerging field of study. Still, recent evidence suggests that the gene is required for intracellular signaling between integrin–focal adhesion and the extracellular matrix, which is proven to be an essential regulator of OFT development [81].

A single-cell RNA sequencing study of CNCCs from Tbx1-deficient mice revealed disrupted signaling pathways that resulted in abnormal aortic arch artery formation and outflow tract septation. Additionally, the loss of *Tbx1* also altered *Tbx2* and *Tbx3* expressing CNCCs, which is problematic because the inactivation of these genes was found to result in failed smooth muscle differentiation, leading to aortic arch branching defects [82]. Clearly, *Tbx1* is not the only T-box gene expressed in the second heart field. In fact, *Tbx5* is expressed in the posterior second heart field, and the encoded protein has been shown to interact with Odd Skipped Related Transcription Factor 1 (Osr1) to aid in the regulation of posterior SHF cell cycle progression [83]. MEF2 also plays a critical role in second heart field development. A deletional analysis in the mouse embryo revealed that the anterior heart field regulatory region of *mef2c*, controlled by an intronic enhancer, is dependent for its activity on both Islet1 and zinc-finger transcription factor GATA4 both in vitro and in vivo [84], and the loss of *mef2c* expression in the SHF driven by this enhancer resulted in severe outflow tract alignment defects [85].

Additional genes involved in SHF formation include *Hes1* and *Pitx2*. The latter is a homeobox gene known to play a pivotal role in cardiac left–right asymmetrical morphogenesis [86,87]. In 2006, a combination of fate mapping and conditional loss of function assays in mice revealed that a deficiency of *Pitx2* in the SHF resulted in severe outflow tract defects. This suggested that *Pitx2*, a regulator of the Wnt-signaling pathway, functions within SHF-derived cells to asymmetrically pattern the outflow tract myocardium [88]. *Hes1*, a transcription factor downstream of the Notch signaling pathway, is also expressed in the mouse SHF progenitor cells. At mid-gestation, *Hes1* homozygous mutant mice have a shorter than average outflow tract due to impaired SHF proliferation, although progenitor cell differentiation is undisturbed [89]. Basic helix–loop–helix (bHLH) transcription factors also play a role, as SHF-specific embryonic knockouts of *Hand2* display defective myocardium and interventricular septum, and the lineage tracing of both *Hand1* and *Hand2* has shown that these bHLH factors are required for normal left ventricle morphogenesis [90,91].

To gain greater insight into SHF developmental processes, single-cell RNA sequencing of separated first and second heart field cells has been carried out. Xiong et al. [92] separately analyzed single-cell transcriptomes from *Nkx2*.5:tdTomato-embryos (labeling both heart fields) compared with cells from Isl-1:tdTomato embryos (labeling SHF cells), and they used a computational approach to distinguish FHF cells from those of the SHF. Subsequent studies have analyzed single-cell transcriptomics from multiple heart populations across several developmental embryonic timepoints [93,94,95].

The Xiong et al. studies [92] identified signaling pathways differentially expressed between the FHF and SHF. WNT signaling from the FHF to the SHF is required for the proliferation of second heart field cells, which is then required for their differentiation into their distinct cell pool [96]. Wnt signaling factors are also responsible for the proper formation of the right ventricle, which is possibly correlated with a downstream BMP signaling cascade [97]. The Wnt/ß-catenin pathway is also known to modulate Isl1 cardiovascular progenitors with ectopic activation in the SHF leading to OFT morphological defects characteristic of congenital heart disease [98].

In addition, several impactful studies presented evidence that autocrine FGF signaling is necessary for SHF morphogenesis. When FGF8, a WNT signaling target, is absent in the early mouse embryo, SHF proliferation is disrupted and heart tube elongation falters [89,99]. Likewise, FGF10 is also expressed in SHF cells and plays a role in promoting proliferation, and there is evidence to suggest that *FGF10* transcription is regulated by key SHF factors *Nkx2.5*, Islet1, and Tbx1, the foremost playing a repressive role [100]. The mechanistic pathways by which FGF-dependent outflow tract development operates are continuing to be explored, but it is understood that it is partially mediated by adaptor protein FRS2a, which acts by linking FGF receptor kinases to downstream signaling pathways. When the expression of *Frs2a* is ablated in the mesodermal cells from the SHF in mice, the result is OFT misalignment and hypoplasia as well as defects in cell recruitment to the OFT, including the previously discussed CNCCs [101].

Taken together, these studies underline the highly interactive processes that occur between the FHF and the SHF during cardiac development and identify a complex genetic network for SHF development, which is still being uncovered.

## 6. Congenital Heart Diseases Arising from Defects in the SHF

Cardiac outflow tract defects and abnormalities are estimated to contribute to at least 30% of known CHDs [102]. Given the role of the SHF in the formation of the outflow tract, right ventricle, and partial atria, researchers have been searching for definitive links between improper SHF development and CHDs. One condition closely associated with SHF malformation is DiGeorge syndrome (DGS), which is a congenital disorder resulting from a microdeletion on chromosome 22 at a chromosomal location termed 22q11.2. The autosomal dominant disorder is a result of a failure of the pharyngeal pouches to fully develop in the fetus, which can lead to the disturbed development of the ears, jaw, tonsils, thyroid, and thymus, as well as the aortic arch and cardiac outflow tract. As a consequence, DGS is associated with numerous conotruncal cardiac abnormalities, such as interrupted aortic arch, truncus arteriosus, Tetralogy of Fallot, and ventral septal defects, among others [103]. The mortality rate of DGS is unclear due to inconsistent diagnosis, particularly in children of African-American origin [104]. Nevertheless, it has been concluded that those with the congenital heart defects previously outlined are at a higher risk of death than those without [105,106].

In 1999, researchers engineered a 1 megabase chromosomal deletion in the mouse, termed Df1, which is syntenic to the region of 22q11.2 commonly deleted in DGS. Heterozygous deletion (i.e., *Df1/+*) resulted in cardiac abnormalities consistent with those seen in humans with heart defects caused by DGS [107]. One of the genes included in the deleted region, the T box transcription factor gene *Tbx1*, had previously been implicated as a candidate for abnormalities caused by DGS due to its apt placement within the DGS chromosomal region and its expression in the embryonic pharyngeal pouches [108]. Researchers generated mice heterozygous for a *Tbx1* null mutant allele (named *Tbx1*^tm1Pa^), and heterozygotes for this mutation showed minor aortic arch abnormalities. However, homozygous *Tbx1*^tm1Pa^/*Tbx1*^tm1Pa^ mice were unable to survive to adulthood. The dissection of homozygous late-term embryos and neonatal mice revealed several cardiac deformities, namely missing outflow tracts and abnormal aortic arches. Thus, heterozygous deficiency of the single *Tbx1* gene results in minor phenotypes mirroring those seen in DGS, while homozygous deletions result in a more severe reflection of the syndrome. This important finding led researchers to the conclusion that the essential SHF gene, *Tbx1*, is the primary gene whose haploinsufficiency causes cardiac defects in DiGeorge patients [47,109,110]. However, since haploinsufficiency for *Tbx1* alone does not recapitulate the severity and frequency of the phenotypes observed in *Df1/+* heterozygotes, researchers have been searching to identify additional genes in the DGS region, as well as unlinked genes, that enhance this effect. Other proteins translated in the vertebrate SHF may contribute to the cardiac defects found in DGS, such as Wnt5a [111], Wnt11-related [112], and GATA4 [113].

## 7. Does Drosophila Have a SHF?

Recent evidence suggests that the ventral longitudinal muscles (VLMs) of the Drosophila linear heart tube may be a valuable model for studying the mammalian SHF. VLMs are densely packed fibers that form ventrally to the cardiac tube during the pupal stage of development when the heart tube is remodeled from the larval structure to that of the adult [28,34]. VLMs were believed to be formed from a subset of lymph gland cells [114], but they were later attributed to the dedifferentiation of alary muscles into mononuclear mesenchymal cells [35]. Given the genetic similarities between mammals and fruit flies, it is no surprise that Drosophila share many of the genes associated with the SHF. As mentioned above, *org-1*, the Drosophila ortholog of *Tbx1*, is expressed in the alary muscles during embryonic development [33]. An ex vivo imaging study identified *org-1* as an essential factor for the lineage reprogramming that transforms alary muscle-derived cells into the longitudinal fibers [35]. Additionally, *tailup* (*tup*), the Drosophila *Islet1* ortholog, is essential for normal heart formation in the embryo [115,116]. Org-1 binds to *Tup-ADME*, an enhancer element, to regulate *tup* expression to control embryonic alary muscle development [117]. This suggests that the *tup-org-1* genetic pathway is re-used in heart remodeling, and moreover, it mirrors the relationship between their vertebrate orthologs, *Tbx1* and *Isl1*. Immunofluorescence staining revealed that both *org-1* and *tup* are expressed throughout the adult ventral longitudinal muscle development, and the induction of RNAi against either *tup* or *org-1* leads to a complete loss of adult VLMs [35]. These studies revealed a conserved genetic circuit required for the formation of the Drosophila ventral longitudinal muscles. We note that the physiological functions of the SHF and the ventral longitudinal muscles are divergent—for example, the VLMs are thought to be modified skeletal muscles rather than cardiac muscle [36,114]—nevertheless, it is feasible that the regulatory circuitry that gives rise to these muscle types has been retained.

Are other orthologs of SHF genes also required for ventral longitudinal muscle development? In a brief survey, Schaub et al. [35] showed that the formation of VLMs is dependent on the FGF receptor Heartless (Htl), MEF2, and the Ecdysone Receptor (EcR). The requirements for FGF signaling and MEF2 function parallel the requirements for their orthologs in vertebrate second heart field development.

## 8. Summary and Future Potential

These tantalizing results support the concept that the Drosophila VLMs are analogous to the SHF, given that a number of orthologous genes are required for their formation in the respective organisms. Nevertheless, the list of Drosophila genes required for VLM formation is relatively small at this point, and a more robust test of this model will require assessment of the contributions of additional loci. Reagents to achieve this are readily available: Gal4 drivers, for inducible gene expression [118], have been generated that function in both the cardiac tube or cardiomyocytes (*Hand–Gal4* and *Sur–Gal4* [119,120] and the ventral fibers (*org-1–Gal4* [35]); and inducible RNAi lines for most genes in the Drosophila genome have been generated [121]. These regents could be applied to a list of genes known to contribute to vertebrate SHF development to test the overall hypothesis. Along these lines, in Table 1, we list a selection of transcription factors known to accumulate in the SHF and their Drosophila orthologs. In addition, this knockdown approach could be applied to candidate genes identified through human sequencing studies, as seen in Ekure et al. [60]; or the approach could be applied in a non-biased way as a means of gene discovery through genome-wide screens to identify the roles of transcription factor or signaling pathways in VLM formation.

A significant advantage to the use of Drosophila in this context is that the VLMs are largely dispensable for adult viability [35], meaning that even serious defects in VLM formation will not prevent the generation of adults for analysis. Moreover, since it is possible to perform a detailed analysis of adult heart physiological parameters in Drosophila [123], it should also be straightforward to define the requirement for these fibers in adult heart function.

In summary, the Drosophila system has made critical contributions to our understanding of mammalian heart development and disease, and this is despite significant differences in the complexity of the cardiac organ from flies to vertebrates. We propose that the Drosophila system can continue to contribute to understanding vertebrate heart development, through studying specification and development of the ventral longitudinal muscle fibers as a model for the formation of the mammalian second heart field.

## Figures and Tables

**Table 1:** Mammalian SHF transcription factors and their Drosophila orthologs. Shown is a non-exhaustive list of differentially enriched transcription factors of the mammalian second heart field along with their Drosophila counterparts. Mouse SHF transcription factors were identified in Xiong et al. [92] using single-cell transcriptomics, and results were finalized using the R package Seurat. Orthologs were identified at http://www.flyrnai.org/diopt (accessed on 5 December 2023) [122], which collates ortholog predictions from a number of online tools. The DIOPT scores indicated show the number of algorithms that predict a gene–pair relationship out of a total of 24 algorithms.

Mammalian Transcription Factor	Drosophila Transcription Factor	DIOPT
Isl1	tup	17
KDM5A	lid	15
WDHD1	Ctf4	14
SMARCC1	mor	14
GLI3	ci	13
TBX1	org-1	12
MIER1	CG1620	12
ARID4B	htk	12
PRDM1	Blimp-1	11
E2F3	E2f1	11
TRP53	p53	11
SALL4	salr	10
TGIF1	achi	10
TCF21	HLH54F	9
LITAF	CG13510	9
IRX5	ara	7
Zfp445	ush	2
ZFP606	crol	2
ZFP710	CG12299	2
Zfp57	sqz	1

## Data Availability

Not applicable.
